# Characterization and Classification of Electrophysiological Signals Represented as Visibility Graphs Using the Maxclique Graph

**DOI:** 10.3389/fbioe.2020.00324

**Published:** 2020-04-15

**Authors:** Erika Elizabeth Rodriguez-Torres, Ulises Paredes-Hernandez, Enrique Vazquez-Mendoza, Margarita Tetlalmatzi-Montiel, Consuelo Morgado-Valle, Luis Beltran-Parrazal, Rafael Villarroel-Flores

**Affiliations:** ^1^Área Académica de Matemáticas y Física, Universidad Autónoma del Estado de Hidalgo, Pachuca, Mexico; ^2^Centro de Investigación y de Estudios Avanzados del Instituto Politécnico Nacional, Mexico City, Mexico; ^3^Centro de Investigaciones Cerebrales, Dirección General de Investigaciones, Universidad Veracruzana, Xalapa, Mexico

**Keywords:** visibility graphs, graph theory, maxcliques, electrophysiological signals, deep learning, pre-Bötzinger complex, XII nerve, sigh

## Abstract

Detection, characterization and classification of patterns within time series from electrophysiological signals have been a challenge for neuroscientists due to their complexity and variability. Here, we aimed to use graph theory to characterize and classify waveforms within biological signals using maxcliques as a feature for a deep learning method. We implemented a compact and easy to visualize algorithm and interface in Python. This software uses time series as input. We applied the maxclique graph operator in order to obtain further graph parameters. We extracted features of the time series by processing all graph parameters through K-means, one of the simplest unsupervised machine learning algorithms. As proof of principle, we analyzed integrated electrical activity of XII nerve to identify waveforms. Our results show that the use of maxcliques allows identification of two distinct types of waveforms that match expert classification. We propose that our method can be a useful tool to characterize and classify other electrophysiological signals in a short time and objectively. Reducing the classification time improves efficiency for further analysis in order to compare between treatments or conditions, e.g., pharmacological trials, injuries, or neurodegenerative diseases.

## 1. Introduction

To understand brain functioning neuroscientists use electrophysiological techniques (e.g., macro-patch and patch-clamp recordings) to assess activity of neurons. Whereas, sharp-electrode and patch-clamp techniques are used to record the activity of a single neuron, extracellular field recordings and macropatch techniques allow recording the activity of many neurons within a population. Macropatch suction electrodes are widely used to record motor nerve activity. The inspiratory phase of the respiratory rhythm is generated in the pre-Bötzinger complex (pre-BötC), a neuronal network in the ventrolateral medulla. In an *in vitro* preparation containing the pre-BötC, inspiratory-related motor output can be recorded from the XII nerve. Nerve activity is integrated and used to classify and characterize the inspiratory-related burst. Frequently, researchers made this manually; however, this is a time-consuming and very subjective task. Spike sorting, traditionally, is made measuring properties of the waveform (e.g., peak latency, spike half-width, amplitude), determining which of these properties or features are relevant (e.g., principal component analysis) and performing cluster analysis (Rey et al., 2015). In the literature, one can find several algorithms employed for the spike sorting, following different steps and approaches. For instance, some of the techniques are based on wavelets, or combinations of wavelets and different approaches of principal components, for a review one can see Rey et al. (2015) and Lefebvrea et al. (2016). A more recent approach is based on the shape, phase, and distribution features of each spike and a clustering algorithm based on k-means (Caro-Martín et al., 2018). Along with the spike sorting algorithms, methods that validate them are necessary (Einevoll et al., 2012). However, in the field of respiratory rhythm there are not automated methods for the identification of sighs. In plethysmographic recordings, sighs are identified visually by the expert. In electrophysiological recordings from reduced preparations *in vitro*, the criteria for defining a sigh are determined by the researcher and therefore vary between research groups. Some groups consider the amplitude as a relevant parameter (Lieske et al., 2000; Lieske and Ramirez, 2006a,b; Ruangkittisakul et al., 2008); others the presence of biphasic burst (Kam et al., 2013; Li P. et al., 2016). Here, based on our analysis we propose to use graph theory to characterize and classify waveforms within biological signals using maxcliques as a feature for a deep learning method.

A network or graph is one of the most intuitive, explicit and clear representation of a complex system. Such graphs consist of *nodes* and *links* representing the participating elements and the interactions among them. Therefore, graphs characterize the structure of complex systems and how its elements interact. That is, they can reflect the dynamics or functions of the complex system if states and transitions are represented by nodes and links, respectively (Gershenson and Niazi, 2013). If one can understand the relationship between structure and function, then the characterization and classification of complex systems can be studied further.

In this work, each time series is associated with a simple graph called *visibility graph*, as defined in Lacasa et al. (2008) and studied in Lacasa and Flanagan (2015). As a remark, this graphs inherits either the periodicity or the randomness of the original time series. Even more, fractal time series are transformed into scale-free graphs (Lacasa et al., 2008). We aimed to use Graph Theory to characterize and classify visibility graphs using maxcliques as a feature for a deep learning method. Here, we analyzed *in vitro* recordings of XII nerve inspiratory activity to classify sighs and non-sighs waveforms. The visibility graph of a non-sighs shows a simpler structure than sighs. The interest of the authors in sighs is its relevance in preventing lung collapses.

Recently, visibility graphs have been employed to analyze the resulting time series from physiological data as in Hou et al. (2016), Jiang et al. (2013), and Shao (2010), in the analysis of complex networks for cardiorespiratory interactions (Long, 2015) or a modified visibility graph for the suicidal tendency (Bhaduri et al., 2016). However, in these works, the concept of maxcliques from graph theory was not implemented in the characterization and classification of waveforms.

We present a graphical interface, written in Python, that helped in the process of constructing the visibility graph from the time series and determining several parameters of the resulting graph. Python is an open source interpreted programming language. The simplicity of Python syntax makes its code readable and understandable, facilitating its learning. There are several Python libraries, such as pandas, numpy, SciPy, and others that allow the user to process and analyze data easily and quickly. Although a Python package with the implementation of the algorithm described in Lacasa et al. (2008) can be found in García-Herrera (2015), the one showed in this work is more convenient and, as a consequence, easier to visualize. The interface employs two Python libraries: *NetworkX* (https://networkx.github.io/), where several algorithms of Graph Theory have been already implemented, and *matplotlib* (https://matplotlib.org/) for the graphs.

We claim that several aspects of a time series can be deduced from certain parameters of the associated visibility graphs. In this work in particular, that was the case with the maximum degree, the clique number, and the number of cliques. They allowed to tell sighs from non-sighs in the time series obtained in the waveforms from *in vitro* recordings of XII nerve inspiratory activity.

## 2. Materials and Methods

### 2.1. Graph Theory

As a mathematical concept, a *graph* *G* is composed by a set of points denoted with *V*(*G*), and a set denoted by *E*(*G*) whose elements are unordered pairs of elements of *V*(*G*). The elements of *V*(*G*) are called *vertices* or *nodes*, and the elements of *E*(*G*) are called *edges* or *links*. The number of vertices in a graph *G* is called the *order* of the graph *G* and is denoted by |*G*|. If the nodes *v*_1_, *v*_2_ are such that {*v*_1_, *v*_2_} ∈ *E*(*G*), we say that the vertices *v*_1_, *v*_2_ are *adjacent*, and we denote that by *v*_1_ ~ *v*_2_. Given a vertex *v*, the number of vertices adjacent to *v* is called the *degree* of *v*. As a starting point for the concepts from graph theory, we recommend Harary (1969) and McKee and McMorris (1999).

**Figure 1:**
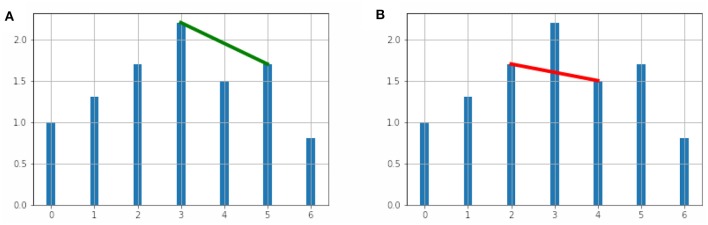
**(A)** The vertices (3, 2.2) and (5, 1.7) are adjacent in the visibility graph. **(B)** The vertices (2, 1.7) and (4, 1.5) are not adjacent in the visibility graph.

Lacasa et al. (2008) associated for the first time a graph to a given time series by a procedure they called the *visibility algorithm*, which we now describe. Given a time series with data pairs {(*t*_*a*_, *y*_*a*_)}, they obtain the *visibility graph* of the time series as the graph where the vertex set is the set of all data pairs, and define that the pairs (*t*_*a*_, *y*_*a*_), (*t*_*b*_, *y*_*b*_) are adjacent whenever we have:

(1) $$\begin{array}{l} y_{c}<y_{b}+\left(y_{a}-y_{b}\right)\frac{t_{b}-t_{c}}{t_{b}-t_{a}}, \end{array}$$

for all data pairs (*t*_*c*_, *y*_*c*_) with *t*_*a*_ < *t*_*c*_ < *t*_*b*_. The geometric visualization of this condition is shown in Figures 1A,B.

Given a graph *G*, a *maxclique* *C* is a subset of its nodes such that every two nodes in *C* are adjacent, and there is no vertex in *G* not in *C* that is adjacent to all the vertices of *C*. We follow McKee and McMorris (1999) in the use of the term “*maxclique*,” in order to avoid the ambiguity found in the literature on the meaning of the word “clique.”

The *maxclique graph* is the graph that has as vertices the maxcliques of *G*, and where two maxcliques *C*_1_, *C*_2_ are adjacent whenever there is at least a vertex of *G* that belongs to both *C*_1_ and *C*_2_. As a reference for maxclique graphs we mention Szwarcfiter (2003). The maxclique graph of *G* will be denoted as *K*(*G*). It follows then that a graph *G* has |*K*(*G*)| maxcliques.

We now define further parameters of a graph *G* that will be considered in this work:

- **Maximum degree:** This is denoted by Δ(*G*), and is the maximum among all degrees of vertices of *G*.
- **Clique number:** This is the number of elements of the largest maxclique of *G*. It is denoted by ω(*G*).

As an example of the concepts described here, consider the time series given by

(2) $$\begin{array}{l} \left[\left(0,1\right),\left(1,1.3\right),\left(2,1.7\right),\left(3,2.2\right),\left(4,1.5\right),\left(5,1.7\right),\left(6,0.8\right)\right] \end{array}$$

In Figure 1A, we show two vertices adjacent in the visibility graph and in Figure 1B we show two non-adjacent vertices.

**Figure 2:**
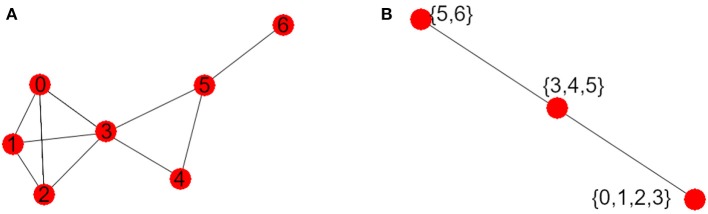
**(A)** Visibility graph *G*. **(B)** Maxclique graph *K*(*G*).

The visibility graph *G* of this time series is shown in Figure 2A. The vertex with maximum degree is the vertex 3, and its degree is 5, and so Δ(*G*) = 5. The graph *G* has three maxcliques, so that |*K*(*G*)| = 3. The three maxcliques are: {0, 1, 2, 3}, {3, 4, 5} and {5, 6}, with 2, 3, and 4 vertices each. Since the greatest maxclique of *G* has four elements, we obtain that ω(*G*) = 4. Finally, note that the second clique intersects each of the other two, and the first and the third do not intersect. So the graph *K*(*G*) has three vertices, as it is shown in Figure 2B.

### 2.2. Interface to NetworkX in Python

**Figure 3:**
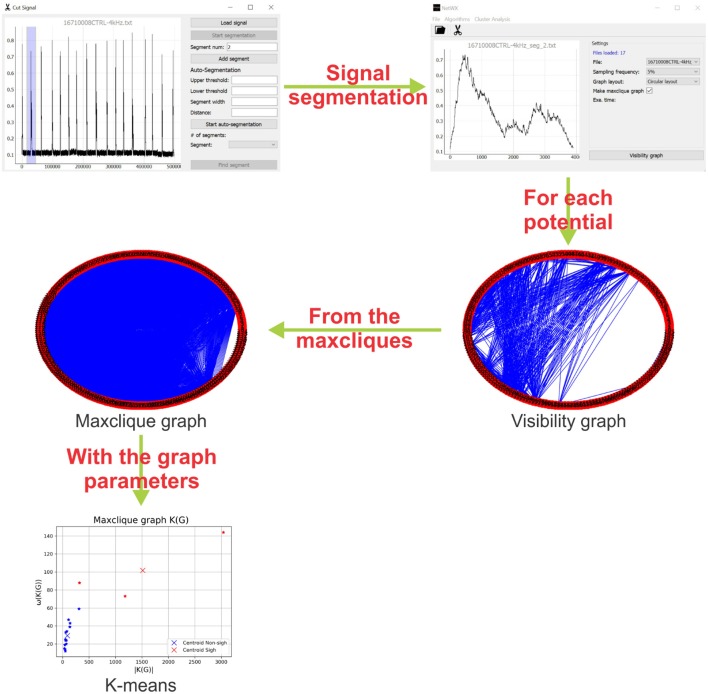
Schematic representation of the methodology. First, the interface identify and segment each potential of the electrophysiology recording. Then, a visibility graph is created for each potential, for large signals a reduction of the sampling frequency is recommended. After that, from the maxcliques determined of the visibility graph, the maxclique graph is created and its parameters are estimated. Finally, a K-means clustering is performed on the maxclique graph parameters. In this work, the result is a classification of the potentials as sighs or non-sighs.

The graph algorithms described in section 2.1 were implemented in a Python interface using the PyQT5 library. The supported files are of one or two columns (*.txt* or *.csv* format). One can select the percentage of sampling frequency (recommended for large signals), visibility graph style and an option to create the maxclique graph (Figure 3).

With the signals loaded and setting the parameters, the visibility graph *G* is created. The visibility graph *G*, the maxclique graph *K*(*G*) (in format *.png*) and the parameters that are calculated in each algorithm (in format *.txt*) are saved to the signals folder. The interface has also a tool to segment or auto segment signals (Figure 3). The button *Start segmentation* enables a bar to select a region in the signal loaded. For auto segment signals the user must introduce an upper threshold, lower threshold, segment width and distance between spikes. In Figure 3, we show a schematic representation of the process to classify electrophysiological signals using maxclique graph parameters.

### 2.3. Experiment

**Figure 4:**
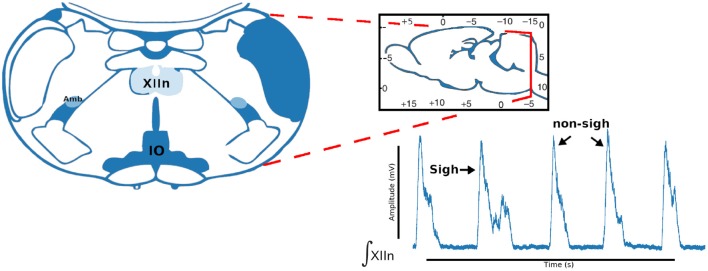
Coronal brainstem section that presents the anatomical marks to locate the pre-Bötzinger complex. Representative integrated activity of the XII nerve showing characteristic waveform of sigh and non-sigh.

The pre-BötC (pre-Bötzinger complex) is a heterogeneous network of interneurons. In rats this contains a population of ~1,000 neurons. In synaptic interactions between pre-BötC neurons each neuron produces inspiratory rhythmic activity in the form of synchronous depolarization of 10–20 mV with a duration of 0.3–0.8 s and with waveforms called inspiratory bursts. In addition to its role in the generation of the respiratory rhythm, pre-BötC is essential for the formation of the respiratory pattern. The protocol for obtaining respiratory rhythm records consists in sectioning the brain stem of neonatal rats under the microscope until the ambiguous nucleus and the inferior olive appear (Figure 4).

**Figure 5:**
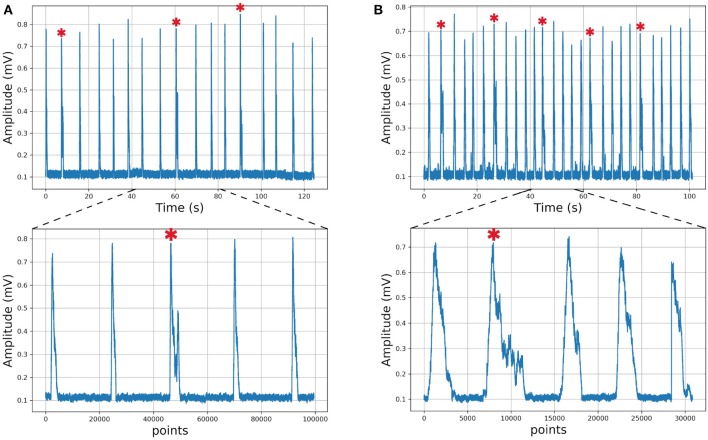
Respiratory rhythm. **(A)** Control record and **(B)** Bombesin record. In asterisk (^*^) are shown sighs.

We describe the electrophysiology in brief. Coronal sections were cut (500–600μm) and the rhythmic activity was recorded from the roots of the XII nerve (XIIn). Then the signal of the XIIn motor neurons excited by pre-BötC neurons is transmitted, obtaining the rhythmic activity of the XIIn (Figure 4). Once baseline activity was established, drug application was performed in the slice bath. In each experiment, two time series were obtained, the first corresponding to control respiratory activity (Figure 5A) and the second when the pre-BötC slice was exposed to bombesin (Figure 5B). In Figure 4 we can observe two components: normal respiratory rhythm (non-sigh) and long inspirations known as sighs. Sighs are biphasic inspiratory bursts. However, sighs can fulfill important regulatory functions. More specifically, a sigh acts as a general restorative of the respiratory system (Patroniti et al., 2002). In general, the pre-BötC generates a normal inspiratory burst every 7–8 s (non-sigh) and every 30–40 s generates a disturbance called a sigh. For more information on how the experiment was done see Munoz-Ortiz et al. (2016).

### 2.4. Statistical Analysis

Given that data did not follow a normal distribution (Shapiro-Wilk test), the Box-Cox transformation was used, as implemented in the R package fpp. With that, λ = −0.475 was determined as the value that maximized the log-likelihood function and yield the best transformation to normality. Some parameters of the visibility graph *G* associated to the time series (Δ(*G*), ω(*G*), and |*G*|) and of the maxclique graph *K*(*G*) (Δ(*K*(*G*)), ω(*K*(*G*)), and |*K*(*G*)|) were compared between sigh and non-sigh using a two-way ANOVA, followed by a Bonferroni's multiple comparisons test. To evaluate the performance of classification based on visibility or maxclique graph parameters, we compared the number of sighs and non-sighs identified by the three classifiers performing a chi-squared test and a pairwise comparison with Bonferroni's correction. Then, we compared both classifications vs. the classifications based on an expert determining the number of successes and failures of each classification. Then, we performed a McNemar's test. Two-way ANOVA was performed in GraphPad Prism (v. 6.00, GraphPad Software, Ca, USA). Box-Cox transformation, chi-squared and McNemar's tests were performed in R (v. 3.6.1—“Action of the Toes”). Significant differences were considered at *P* ≤ 0.05. Data is showed as mean ± S.E.M.

## 3. Results and Discussion

### 3.1. Results

As an example of usefulness, we employed *in vitro* recordings from XII nerve respiratory rhythm activity of rats in order to obtained time series describing burst amplitude. In this time series, we can differentiate between sigh and non-sigh waveforms, which were recorded in control and bombesin conditions. First of all, we wanted to determine if the classification between sigh and non-sighs was correct, independently of the experimental condition. To achieve the latter, we used a short time series composed of 17 potentials of control recording (Figure 5A), which were previously classified by an expert in 14 non-sighs and 3 sighs. Likewise, we used a bombesin recording composed of 27 inspiratory bursts (Figure 5B), 22 non-sighs and 5 sighs.

**Figure 6:**
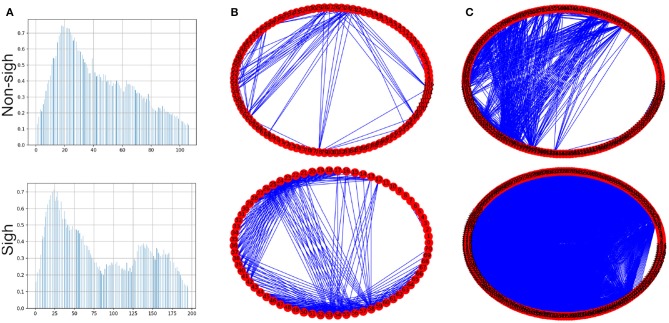
**(A)** Inspiratory burst recordings with its sampling frequency reduced to 5% of non-sigh and sigh time series from respiratory rhythm *in vitro* recordings. **(B)** Circle visibility graphs constructed from time series shown in **(A)**, for non-sigh *G*_1_ and sigh *G*_2_, respectively. The non-sigh circle visibility graph may appear to show fewer connections than the sigh one. **(C)** Maxclique graphs for non-sigh and sigh. In this case, it is apparent that the number of connections (that is, edges) is much larger for sigh (*K*(*G*_2_)) than non-sigh (*K*(*G*_1_)).

To create the visibility graphs the sampling frequency of each inspiratory burst was reduced to 5%, in both sighs and non-sighs waveforms (Figure 6A). The visibility graph of the non-sigh and sigh will be denoted by *G*_1_ and *G*_2_ (Figure 6B), respectively. Now, for each visibility graph, *G*_1_ and *G*_2_, we constructed their maxclique graphs, denoted as *K*(*G*_1_) and *K*(*G*_2_) (Figure 6C), respectively. From both graphs, we calculated their maximum degree Δ(*G*_1_), Δ(*G*_2_), clique number ω(*G*_1_), ω(*G*_2_), and number of cliques |*K*(*G*_1_)|, |*K*(*G*_2_)|.

Classification of waveforms was performed using K-means clustering analysis with the three graph parameters [clique number: ω(*G*), number of maxcliques: |*K*(*G*)|, and maximum degree: Δ(*G*)] of each graph [visibility, *G* and maxclique, *K*(*G*)], comparing in pairs. Of these parameters, we observed that clique number and number of maxcliques classify better both waveforms, independently of experimental condition.

**Figure 7:**
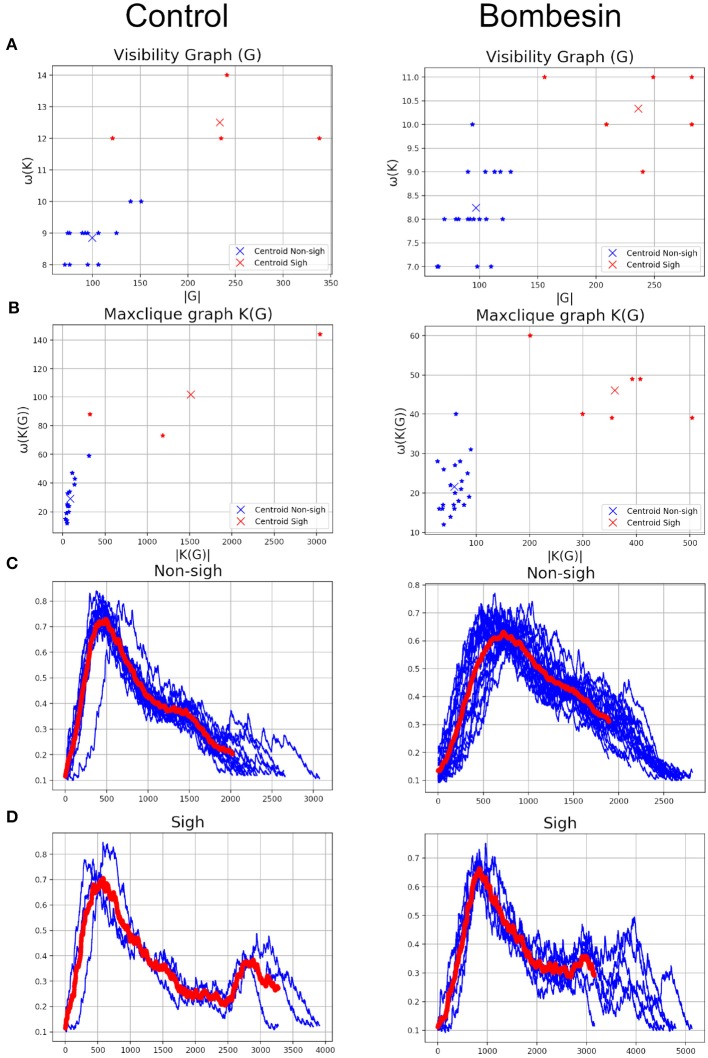
Classification according with clique's parameters. Control on first column and bombesin recording on second column. **(A)** Visibility graph and **(B)** Maxclique graph K-means cluster analysis, **(C)** non-sighs, and **(D)** sighs inspiratory bursts. In red are shown the means of all inspiratory bursts classified with Maxclique graph.

K-means clustering analysis with visibility graph parameters resulted in 13 non-sighs and 4 sighs in the control recording, and 21 non-sighs and 6 sighs in the bombesin recording (Figure 7A). In contrast, K-means clustering analysis with maxclique graph parameters resulted in 14 non-sighs and 3 sighs in the control recording, and 21 non-sighs and 6 sighs in the bombesin recording (Figure 7B). In Figures 7C,D, we show the inspiratory bursts as classified by the maxclique graph parameters, in both control and bombesin condition, which shows that this classification is accurate. Altogether, these results show that the clique number and the number of max cliques of the maxclique graph have a better classifying waveforms performance.

**Figure 8:**
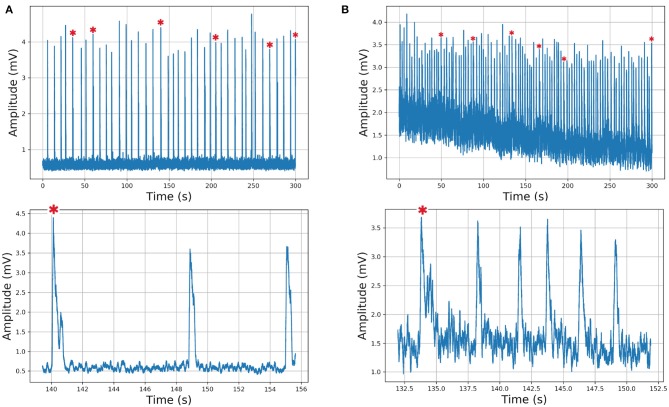
Respiratory rhythm recording and corresponding visibility graphs for **(A)** control with 39 and **(B)** bombesin with 99 inspiration burst. In asterisk (^*^) are shown sighs.

In the previous description, we used an expert delimited and classified waveforms. However, we created an automatic segmentation and performed the same analysis to evaluate if the classification remained consistent. In this case, we used a time series composed of 39 and 99 inspiratory bursts, recorded in control and bombesin conditions, respectively (Figure 8).

**Figure 9:**
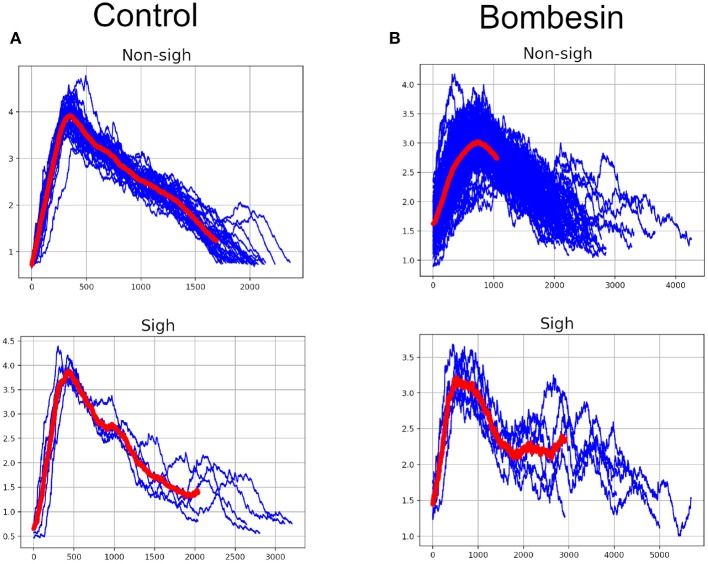
Inspiratory burst classification according with clique's parameters in Maxclique graph. **(A)** Control with 39 inspiratory burst and **(B)** bombesin with 99 inspiratory burst. In red are shown the means of all inspiratory bursts classified.

The automatic segmentation identified every single burst. Classification based on visibility graph parameters resulted in 10 sighs and 29 non-sighs, in the control recording and 42 sighs and 57 non-sighs, in the bombesin recording. On the other hand, classification based on maxclique graph parameters resulted in 5 sighs and 34 non-sighs, in the control recording and 6 sighs and 93 non-sighs, in the bombesin recording. The inspiratory bursts as classified by the maxclique graph parameters, in both control and bombesin condition, are shown in Figures 9A,B, respectively. This suggests that automatic segmentation properly identifies potentials, regardless of the waveform and experimental condition.

**Figure 10:**
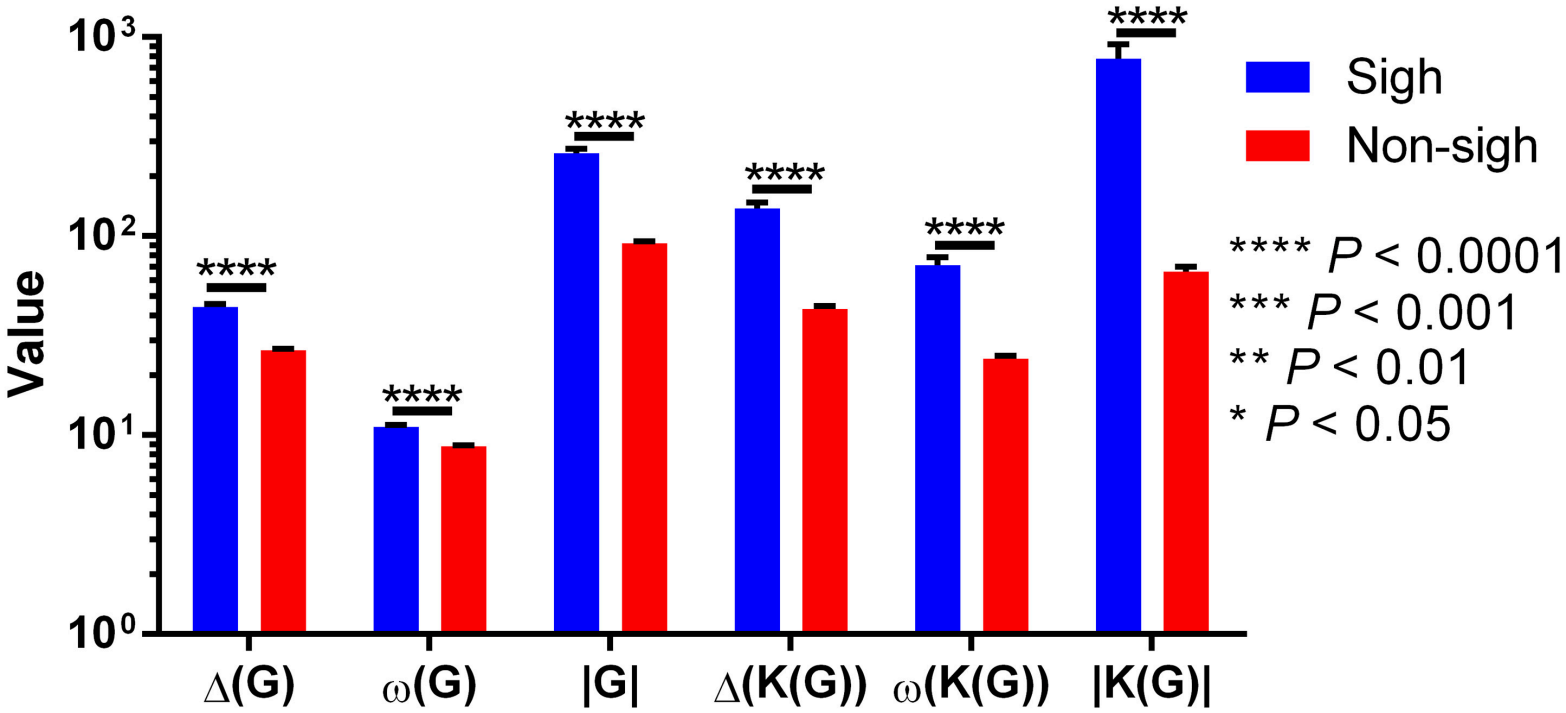
Visibility (Δ(*G*), ω(*G*), and |*G*|) and maxclique (Δ(*K*(*G*)), ω(*K*(*G*)), and |*K*(*G*)|) graph parameters of sigh and non-sigh waveforms. Data is showed as mean ± S.E.M. Significant differences between non-sigh and sigh were determined using a two-way ANOVA, followed by Bonferroni's multiple comparisons. ^*^P < 0.05, ^**^P < 0.01, ^***^P < 0.001, ^****^P < 0.0001. Sighs, *n* = 20; non-sighs, *n* = 162.

On previous results, we observed that maxclique parameters seem to classify more accurately between both waveforms. Thus, in order to determine if this is robust enough, we performed the analysis with a larger time series, composed of 182 potentials. After K-means classification based on visibility or maxclique parameters we compared between putative sigh (20 potentials) and non-sigh (162 potentials) waveforms. Our analysis showed that both visibility and maxclique graph parameters show statistical difference between sigh (S) and non-sigh (NS) (graph parameters, *F*_5, 1080_ = 579.2, *P* < 0.0001; waveform, *F*_1, 1080_ = 508.4, *P* < 0.0001; graph parameter^*^waveform, *F*_5, 1080_ = 14.66, *P* < 0.0001). Bonferroni's *post-hoc* test showed that *G* max degree (S, 44.10 ± 1.60 vs. NS, 26.62 ± 0.48; *P* < 0.0001; Figure 10), *G* clique num (S, 11.00 ± 0.27 vs. NS, 8.77 ± 0.10; *P* < 0.0001; Figure 10), *G* number of max cliques (S, 260.60 ± 14.01 vs. NS, 92.10 ± 2.21; *P* < 0.0001; Figure 10), *K*(*G*) max degree (S, 137.40 ± 10.26 vs. NS, 43.10 ± 1.42; *P* < 0.0001; Figure 10), *K*(*G*) clique num (S, 71.55 ± 6.69 vs. NS, 24.25 ± 0.85; *P* < 0.0001; Figure 10), and *K*(*G*) number of max cliques (S, 779.40 ± 142.80 vs. NS, 66.32 ± 3.94; *P* < 0.0001; Figure 10) differed between sighs and non-sighs. This suggest that the groups generated by the K-means are authentic groups.

However, the above does not imply that these groups represent real sighs and non-sighs. First, we compared the number of sighs and non-sighs classified with both parameters and by an expert, which resulted to be different (χ^2^ = 40.84; *df* = 2; *P* < 0.0001). Our pairwise comparison analysis showed that classification based on visibility graph parameters (S, 61; NS, 162) is statistically different from that performed by the expert (S, 20; NS, 162; χ^2^ = 25.41; *df* = 1; *P* < 0.0001). In contrast, the classification based on maxclique graph parameter (S, 20; NS, 162) did not differ from the classification performed by the expert (S, 20; NS, 162; χ^2^ = 0; *df* = 1; *P* = 1).

Although our previous results showed that maxclique parameters identify the same number of sigh and non-sigh as the expert, we determined the number of successes and failures to assess the accuracy of classification. Our results showed that the classification based on maxclique graph parameters had six failures (three sighs and three non-sighs) and 176 success, whereas classification based on visibility graph parameters had 41 failures (all non-sighs) and 141 successes. McNemar's test showed that maxclique graph parameters were better to correctly identify and classify sigh and non-sigh waveforms (McNemar's χ^2^ = 82.747, *df* = 1, *P* < 0.0001). Altogether, these results indicate that the classification based on maxclique graph parameters is robust to classify accurately between sighs and non-sighs. Also, this suggests that these parameters should be used to classify other waveforms.

### 3.2. Discussion

In this paper, we have presented a classification and characterization of electrophysiological signals using graph parameters applied to visibility graphs and to the result of a graph operator called the maxclique graph, which is denoted by *K*(*G*). The parameter ω(*G*), and the enumeration of the maximal cliques have already been considered in bioinformatics, for example in proteins and genes (see Tomita et al., 2011).

The maxclique graph operator has already been applied to Loop Quantum Gravity (for example see Requardt, 2000). To the best of our knowledge, this is the first time that the maxclique graph operator has been used in electrophysiological signals characterization. We have verified the usefulness of this operator for the task of identifying sighs and non-sighs waveforms, using *in vitro* recordings of XII nerve respiratory rhythm, and implementing in Python an interface using the algorithms described in this work. We think that it is apparent that this software can also be applied to characterize other electrophysiological recordings. The advantage of using cliques is the following:

- As shown in Figures 7A,B, the maxclique graph *K*(*G*) allows us to differentiate sighs and non-sighs better than the visibility graph alone.

These results suggest that maxclique graph (*K*(*G*)), and particularly its parameters of number of cliques (|*K*(*G*)|), and clique number (ω(*G*)) have a better performance characterizing and classifying these electrophysiological signals than a visual inspection of the time series. This is because if the time series has many small fluctuations (like sighs), then the visibility graph will have many small cliques, therefore, the graph parameter |*K*(*G*)| will be relatively big and the parameter ω(*G*) will be relatively small. On the other hand, if in the time series there are few fluctuations and a value of the data much larger than the others, then there will be a big clique in the visibility graph, resulting in a small value of |*K*(*G*)| and a larger value of ω(*G*) (like non-sighs). Sighs, and other breathing patterns are embedded within eupneic (normal breathing) signals. Unbiased detection of patterns is a challenge for electrophysiologist. The use of visibility graphs and maxclique analysis provides a tool for sorting waveforms probing a larger number of parameters, instead of commonly used peak amplitude, burst durations or the presence of biphasic shape.

Our statistical analysis showed that visibility and maxclique parameters differ between sigh and non-sigh. Nevertheless, we need further studies to correlate these parameters with their biological meaning to determine what these differences could mean in physiology. Allowing us to implement these graph parameters to compare between different conditions and treatments.

### 3.3. Conclusion

Applying graph theory to electrophysiological recordings we were able to characterize and classify sighs and non-sighs. The visibility graphs and maximum degree allowed to characterize and classify between sighs and non-sighs. Even though the visibility graphs were not effective, the maxclique graphs and parameters of clique algorithm generated a characterization more effective with more successes. Altogether, these results suggest that maxclique graphs and its parameters are more suitable to characterize and classify electrophysiological signals. Likewise, the graphical interface developed allows applying this methodology to other electrophysiological signals.

## Data Availability Statement

The data employed to support the findings of this study have been deposited in the *github* repository mentioned before, i.e., https://github.com/Ulipaeh/vgraph.

## Ethics Statement

The animal study was reviewed and approved by Norma Oficial Mexicana (NOM)-062-ZOO-1999 NIH Guidelines for the Euthanasia of Rodent Fetuses and Neonates.

## Author Contributions

ER-T, UP-H, EV-M, MT-M, CM-V, LB-P, and RV-F conceived and designed the study, and contributed in typing the manuscript. UP-H produced the graphical interface. EV-M contributed the statistical analysis and writing. CM-V and LB-P contributed the electrophysiological experiments. RV-F applied the graph theory concepts and properties.

### Conflict of Interest

The authors declare that the research was conducted in the absence of any commercial or financial relationships that could be construed as a potential conflict of interest.
